# A Systematic Review on Quadriceps Angle in Relation to Knee Abnormalities

**DOI:** 10.7759/cureus.34355

**Published:** 2023-01-29

**Authors:** Rahul Sharma, Vikas Vaibhav, Raviprakash Meshram, Brijendra Singh, Gitanjali Khorwal

**Affiliations:** 1 Anatomy, All India Institute of Medical Sciences, Rishikesh, IND; 2 Forensic Medicine and Toxicology, All India Institute of Medical Sciences, Rishikesh, IND; 3 Forensic Medicine, All India Institute of Medical Sciences, Rishikesh, IND

**Keywords:** chondromalacia pattela, genu varum, genu valgum, patellofemoral pain syndrome, quadriceps angle

## Abstract

Previous studies on the quadriceps (Q) angle and its relation to knee problems have led to conflicting conclusions. In this comprehensive review, we evaluate recent studies on the Q angle and analyze the changes in Q angles. Specifically, we investigate the variation in Q angles when measured under the following conditions: 1) under various measurement techniques; 2) between symptomatic and non-symptomatic groups; 3) between samples of men and women; 4) unilateral versus bilateral Q angles; 5) Q angle in adolescent boys and girls. It is widely believed that Q angles are more significant in symptomatic patients than in asymptomatic individuals or that the right lower leg and left lower limb are equivalent, which is supported by little scientific data. However, research states that young adult females have higher mean Q angles than males.

## Introduction and background

The quadriceps (Q) angle is formed by the Q line of pull from the middle of the patella to the anterior superior iliac spine [1]. It is possible to calculate the degree of force between the Q muscle group and the tendon of the patella in the frontal plane in the extended knee [2,3]. The average Q angle for men is 14˚ and for women is 17˚; an excess of 15-20˚ in Q angle value is typically considered to contribute to knee extensor dysfunction and patellofemoral pain [4-11]. It is often used as an anatomical risk factor for developing chondromalacia patella [3,12] and patellar subluxation or dislocation [13-18]. However, it is now believed that the angle of the Q is a less reliable physical assessment tool than was previously assumed in lower extremity injuries [19-21].

## Review

Methodology

Data were collected from a variety of scientific sources using electronic databases such as PubMed, Medline, Google Scholar, Google Advance Search, Psyc INFO, ROAJ, DOAJR, PED ro, CINAHL, the Cochrane database, ISI Web knowledge, and Web science. Each relevant article was then critically examined in accordance with the study's objectives.

Selection procedure

**Figure 1:**
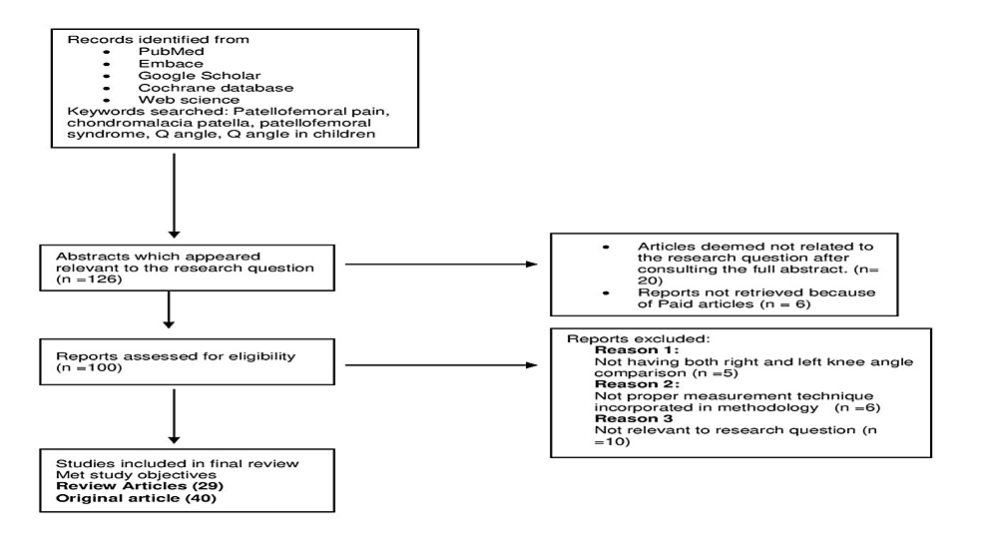
Selection process used for this study as per PRISMA guidelines.

A total of 125 articles were evaluated using the eligibility criteria in the first attempt. The exhaustive selection process used for this study is shown in Figure 1.

Inclusion and exclusion criteria

Research related to the analysis concept was included in this project; however, studies that did not match the objective were excluded from the process.

Measurement methodology and its impact on Q angle magnitude

A line drawn from the midpoint of the patella to the anterior tibial tuberosity and from the midpoint of the patella to the anterior superior iliac spine is used to represent the Q angle [3]. The standard goniometric method is prevalent among medical practitioners, placing individuals in a recumbent supine position with the knee extended and the Q relaxed [22]. However, others see the need to assess the Q angle in situations that more specifically reflect the functional status of the lower limb [23,24], and the pursuit of increased measurement accuracy and reliability [25] has provided the rationale and impetus for methodological change. Contrarily, Q angles have been investigated with people standing [26-29], with their knees flexed and in movement [26,30,31], with the knee extended [25,32], and with uniform foot placement while adopting over ground [25,27,33]. Moreover, using a universal goniometer remains a choice of instrument for many [25,26,34-38], and the application of sophisticated tomography [39] and computed-based video measurement has increased in popularity in recent ages [27,30,31,40].

The lack of a standard measurement method is problematic because it is challenging to compare probes using different approaches directly [25,26]. A slight increase in the Q angle, ranging from 0.3˚ to 1.5˚ [25,38,41] occurs when there is an alter in position from supine to standing. There will be a significant reduction in Q angle from 1.1˚-3.5˚ as the Q contraction moves the patella superiorly and laterally [25,42,43]. Another less-known mechanism is when the lower leg rotates internally over the femur, there is a decrease in Q angle as the knee bends from extension to flexion position [2,3,16,21,39]. Although methodological issues limit the generalizability of this discovery, there is some limited research that indicates the Q angle drops anywhere from 1 to 3 when knee flexion occurs during dynamic activity [26,30,31]. There is limited evidence to support the idea that a change in foot position causes a change in Q angle magnitude. While it is believed that foot position is a factor that needs to be managed during measurement [25,39,44], according to a study, there would be an increase or decrease of angle occurring roughly at 5˚ for every 15˚ internal or external foot rotations [45].

There needs to be more data to accurately estimate variations in Q angle magnitude with changes in the subject position. More research is necessary, particularly studies on how foot location and knee flexion affect the size of the Q angle. If the variation in reported Q angles between studies is believed to represent the significant variation between the samples studied rather than the outcome of the measurement procedure, a standardised measurement technique should also be necessary.

Q angles in asymptomatic vs. symptomatic population

A Q angle above 15˚-20˚ is usually identified as an anatomic risk factor in developing patellofemoral joint pathology. Although little scientific evidence supports this claim, it is assumed to lead to knee extensor dysfunction and discomfort [36]. Although there is limited evidence, it appears that people with chondromalacia patella have excessive Q angles, which are associated with knee deformities [33,46,47]; it is questionable whether those experiencing patellofemoral pain also have such a relation [26,32,36,40,48], recurrent patellar subluxation or dislocation [32,39,46], or other knee and lower leg injuries [37]. Inadequate explanation of data sets, poor data analysis, and inability to jointly collect data from equivalent control groups limit the representativeness of many of the previously concluded initiatives.

Interestingly, Q angle values in patellofemoral disorder symptoms often do not surpass the pathological range of 15-20˚. Some suggest that pathological margins are very generous and propose that they be reduced to somewhere between 15-17˚ [36-39]. However, it does not explain why some individuals with Q angles less than 15˚ endure the deformities mentioned above [26,44, 46,47,49]. Furthermore, research comparing patients with patellofemoral illness who are asymptomatic against those who are symptomatic repeatedly demonstrates that while mean group values can vary greatly, maximum and lowest Q angles exhibit slight variation between groups. At the same time, Caylor et al. [46] did not find significant differences in Q-angle asymptomatic patients (X = 11.1˚) and symptomatic subjects (X = 12.4˚). In this group of patients, increased Q-angles were not the cause of the anterior knee pain syndrome (AKPS). It was also noted that the lowest values for asymptomatic individuals and those with anterior knee pain were -6˚ and 2˚. However, the utmost value was 24˚ for both groups under the weighting situation. As a result, there is much overlap between the Q angle values observed in asymptomatic and symptomatic populations, raising doubts about the standard approach of basing findings primarily on statistical comparisons of group means. As Caspari [50] suggests, rather than treating deviations from the mean as a deviation or disease, it would be more beneficial to identify specific limits of the range of variation in a human trait (such as Q angle).

Overall, there is inadequate data to support the widely accepted theory that having a high Q angle will make someone more likely to have a pathological knee problem. Instead, there is a belief that the extensor mechanism has problems of abnormal Q angle alone [34,37,51]. The quantity and quality of training and the target population's anatomical and physical traits may all impact whether or not the Q angle is linked to a higher level of injury risk [27,32,48].

As potential contributing factors, it is important to take chronic overloading of the knee joint as well as a sudden increase in weekly mileage or exercise intensity into account [14,19,32,36]. Other contributing factors are abnormality of the articular cartilage or subchondral bone [6,9,22,32,46,52], malalignment of lower limb structures [5,13,27,41,51], unequal limb length [12,37], excessive rearfoot motion [30,35,37], muscular and ligamentous insufficiencies [6,14,36], and the carrying of excessive body mass [35].

Q angle in males vs. females

According to research, young adult women have higher mean Q angles than their male counterparts, with disparities ranging from 2.7˚ to 5.8˚ [25,38,46,53] and 3.4˚ to 4.9˚ [25,34,38] when assessed with Q related to supine and standing positions, respectively. Although such studies in patients with patellofemoral dysfunction are uncommon, they show that women typically have Q angles that are 2.0˚ to 8.5 times larger than men [46,47,49]. According to Hvid and Andersen [44], there was a difference of 8.0˚ in mean Q angle values between males and females with patellofemoral diseases. There was no statistically significant difference in mean Q angle between male and female respondents, according to only two groups of researchers [28,41]; nevertheless, larger values were still noted for women. According to any research, young adult males do not show a larger mean Q angle than young adult females.

Considering the variations in the corresponding minimum Q angles for each sex stated, the difference in average Q angles between males and females is expected. Researchers constantly find that healthy females have larger minimum Q angles (2.5˚-10˚) than healthy males (0˚-8.0˚) [25,28,34,38,41]. In contrast, maximum values did not differ significantly from values ranging from 15˚ to 25.5˚ for females and 15˚ to 27˚ for males [25,34,38]. It is unusual to find similar reports of minimum and maximum Q angle values among knee diseases by sex. Although minimum Q angles were similar between the sexes in two of these trials, the results showed that female with patellofemoral pain showed significantly higher maximum Q angles of 29-30˚ than their male counterparts 18˚ [44,47]. Interestingly, these minimum and maximum value patterns are the exact reverses of those observed in people without knee problems.

The differences between the mean, lowest, and maximum Q angle values between the sexes are widely known, but the causes of the differences are less understood. Many claims that women's broader gynaecoid pelvis, as opposed to men's smaller android pelvis, account for the more significant Q angle in women [5,8,11,13,14,39,52]. To restore a mechanical axis through the hip, knee and ankle, a wider pelvis would generate a more lateral proximal reference point for Q angle measurement [47]. It would require a higher valgus orientation of the knee on weight-bearing [5,38,52].

Nevertheless, the idea that women have a broader pelvis than men is challenged by considerable research. Indeed, total pelvic width expressed as a measure of biiliocristal [54], bitrochantric [34], or anterior superior iliac spine breadth [25] are very similar in both sexes. Furthermore, empirical investigations [25,34] could not show a substantial relationship between hip-width and Q-angle measures in men and women. According to Nicola [54], there are probably two factors that have a significant impact, but it is obvious that the relationship between the factors is not understood. These factors are the obvious disregard for the available data on pelvic width and the general inability to distinguish between measurements of absolute pelvic width and a relative value reported as a percentage of height or other widths.

Various literatures postulate alternative justifications as to why females have a raised Q angle than males. Pincivero [34] examined the idea that shorter femurs in women might enhance the valgus of the lower leg and raise the Q angle, expanding on the work of a prior hypotheses. Their results, however, were insignificant. Additionally, it has been suggested that strengthening the Q through exercise and participation in sports may change the degree of the Q angle [43]. Furthermore, an investigation is required to confirm or contradict this idea.

Unilateral vs. bilateral Q angle measure

Researchers frequently record and publish a single Q angle value for each subject [47,49] or group [25-27,33-35,38,40,41,46] they have investigated. This strategy is useful when measurements are restricted to one problematic leg [47,49,55] or the right limb to investigate methodological difficulties [25,38] or when there is no discernible difference in Q angles in the two lower limbs [41,46,53]. There is no adequate justification for reporting a single Q-angle value when the data might have come from either the right or left lower limb, or from both. This is challenging because it restricts the generality of the given conclusions and indicates that Q angles are bilaterally symmetric, a notion that, in the context of the evidence so far, seems controversial.

From a statistical perspective, the findings of the two experiments support the idea of bilateral Q-angle symmetry. Hvid and Anderson [44], in a study of 29 men and women with patellofemoral complaints, reported a mean Q angle value of 16˚ for both the left and right knees. Meanwhile, Lankhorst et al. [36], in a study of 20 patients with patellofemoral pain syndrome (PFPS), showed a more considerable Q angle value of 20˚ compared to the control in one study with PFPS patients. The differences in the results are minimal, but the small sample numbers make it impossible to differentiate between measures taken from limbs with and without symptoms. However, the use of measures of central tendency and conventional analysis of variance models to assess the data is more troublesome because it is recognized that both of these methods can obscure the data's underlying variability [50,56].

While statistical analysis using average data supports the idea of bilateral Q-angle symmetry, individual data analysis supports the contrary; for example, a study done by Jaiyesimi and Jegede [28], showed that in patients with right leg dominance, the right Q angle was more significant than the left. In contrast, in male individuals with left leg dominance, there was a statistically significant difference in the bilateral Q angle. In male subjects, Q-angles were 12.30˚ for the right lower limb and 10.38˚ for the left lower limb, respectively, whereas they were 17.06˚ for the right lower limb and 14.84˚ for the left lower limb in female patients. A case-by-case analysis of the data showed that 10 of 50 participants had a difference in Q angle that varied from 8˚ to 10.3˚. In contrast, nearly half of the subjects had at least four bilateral Q angle differences. A similar bilateral difference in Q angle was observed in the group of non-injured versus sick basketball players, averaging 1.3˚ and 2.7˚, respectively, by Shambaugh et al. [57]. In addition, Mc Connell [58] reported asymmetry in the Q angle of patients who suffered from chondromalacia patellae based on asymptomatic vs. symptomatic and more vs. less symptomatic knee comparison.

There needs to be more information available to state that Q angles are bilaterally symmetric. There can be significant individual variation in the Q angle, and the investigator's statistical approach may have a significant and sometimes, incorrect impact on the findings. Using both grouped and individual observations, the Q-angle data is evaluated.

Q angle in adolescent boys vs. girls

Interestingly, significantly less evidence is present in the literature regarding the Q angle value in adolescent children. Espandar [59] claims that angular malformation of the lower limb is frequent during childhood and is a benign disorder caused by a difference in the typical growth pattern. Chantraine [60] postulated that a growth deformity might be caused by a joint being subjected to a lot of stress and strain during adolescent growth due to extensive sports activity. This possibility is pertinent and has not yet been investigated for the development of angular deformity. Brook and Gross [61] conclude that genu varum is a somewhat common congenital disability in children and will be corrected with growth. By 18 to 24 months, the lower leg gradually straightens with zero tibiofemoral angles due to expected growth (when the newborn begins to stand and walk). By the time a child is seven years old, genu valgum has spontaneously corrected itself to the adult alignment of the lower limb, which is 8 degrees for females and 7˚ for males. With continued normal development, the knee gradually drifts into the valgus (knock-knee) position, reaching its maximum around three to four years with an average lateral tibiofemoral angle of 12˚ [59,61,62].

In comparison, several studies identified the presence of genu varum as a risk factor for developing PFPS [63-67]. Overall, insufficient evidence is available regarding children's average Q angle value; only two studies are present in the literature (Bhalara et al.) which state that the average mean Q-angle value for boys is 15.7±4˚, while the average mean Q-angle value for girls is 15.8±3.4˚ [68]. They also state that as children age, the importance of the Q angle increases significantly, with no discernible differences between the sexes at any age. Cankaya et al. [69] measured the Q angle in healthy children aged between 2 and 8 and found that the average Q angle value for males in a supine position for the right knee was 13.30±1.21˚ and 13.25±1.22˚ for the left knee; whereas the average Q angle value for females was 13.32±1.17˚ for right knee and 13.29±1.14˚ for the left knee in the supine position. In standing position, the average Q angle value in males was 13.27±1.22˚ for right knee and 13.25±1.23˚ for the left knee, whereas in females, the value was 13.30±1.16˚ for the right knee and 13.29±1.18˚for the left knee. This concludes that the Q angle decreases with age in children, regardless of sex, the presence of pes planus, or in the supine and standing position.

There is less significant literature present on Q angle in adolescents. Additionally, there is a need for more significant research on the Q angle in children under the following categories 1. symptomatic and asymptomatic populations of children, 2. unilateral vs. bilateral Q angle measurement, 3. The effect of increased Q angle in children.

Out of the 125 articles evaluated, 69 papers that met the objectives of the study were included (Table 1).

**Table 1:** Studies included in the review. ACL- Anterior cruciate ligament, PFPS- Patellofemoral pain syndrome, PFP- Patellofemoral pain, QF- Quadriceps femoris, IMAEJ- Image processing and analysis in JAVA, VMO- Vastus medialis obliquus, EMG- Electromyogram, TF- Tibiofemoral, ITBFS- Iliotibial band friction syndrome.

S.no	Study author(s)	Year of Publication	Type of study	Sample Size (n)	Conclusion
1.	Loudon JK [1]	2016	Review article	-	Range of magnitude over the patellofemoral joint influence its function and that’s helps to understand the wide variety of clinical problem related to the joint
2.	Boling et al. [2]	2009	Prospective cohort.	1,597	development of Patellofemoral pain is significant related to the weaker hip abductors, knee flexors and Knee extensor strength and increase in Q angle
3.	Mizuno et al. [3]	2000	Descriptive observational	6	Increase and decrease of Q angle affect the positioning of patella and also influence the tibial rotation
4.	Huston et al. [4]	2000	Review article	-	Q angle can change with an isolated Quadriceps contraction , by using athletic training, the dynamic activity could help to lower the Q angle
5.	Frank et al. [5]	2007	Review article	-	Adolescent athlete is susceptible to a variety of injuries and that may differ adult athlete and Qangle should be determined for the patellofemoral syndrome
6.	Kim and Parikh [6]	2022	Review article	-	Patellofemoral instability is common in paediatric knee injuries, which is result from the loss of dynamic relationship of the patella and increase Q angle is one of the risk factor for the development of PFI because it affects patellofemoral kinematics.
7.	Covassin et al. [7]	2014	Cross – sectional study	525	Excess of 15-20˚ in Q angle value is typically considered to contribute to knee extensor dysfunction and patellofemoral pain
8.	Hilibrand et al. [8]	2015	Review article	-	An increased Q-angle is speculated to cause an increase in the lateral pull of the quadriceps, which would place the knee in a vulnerable position for ACL injury and increase valgus stress across the knee
9.	Dixit S et al. [9]	2007	Review article	-	Q angle is one of the anatomical factor in development of PFPS, excessively worn or inappropriate footwear also may contribute in development of PFPS
10.	Grelsamer [10]	2000	Review article	-	Q angle measure the patella tendency to move laterally during quadriceps contraction and a small percentage of patients with patellar pain has increase Q angle
11.	Percy and Strother [11]	1985	Review article	-	Q angle are associated with increase patellofemoral pressures and Malrotation of tibia, such as eternal rotation can also lead to Increase the Q angle
12.	Van Gent et al. [12]	2007	Review article	-	Common site of lower extremity running injuries was knee and greater training distance per week in male runner and history of previous injuries are risk factor for lower extremity running injuries.
13.	Murray et al. [13]	1999	Prospective study	431	sensitivity for patellar pain and patellar dislocation on the axial view was 27% and 62% and for lateral view in full extension was clearly more sensitive for patellar pain (66%) and dislocation (98%). For the flexed-knee lateral view, the specificity (93%) for patellofemoral malalignment was superior to that of the axial view (82%) and the lateral extended view (65%).
14.	Esculier et al. [14]	2020	Review article	-	Quadriceps angle, patellar-tilt angle, sulcus angle, and trochlear inclination in those who eventually develop PFP are no different from those who do not, and healthy knees exhibit a high degree of variability.
15.	Patel et al. [15]	2017	Review article	-	Epiphyseal fracture at distal femur or proximal tibia are seen in younger children, whereas cruciate /collateral ligament and meniscal injury are more common after skeletal maturity is reached; extensor mechanism injury is also common in older adolescents
16.	Fulkerson [16]	2002	Review article	-	Using neyret technique to establish a tomographic Q angle helps clinician to establish incongruities and excessive lateral alignment factors radiographically
17.	Steinberg et al. [17]	2017	Prospective Observational	271	Decreased flexibility of the hamstring and quadriceps muscles were found to expose athletes to a higher risk for subsequent muscle injury in general and to the development of greater patellar tendonitis in particular
18.	Waterman et al. [18]	2012	Retrospective study	40,544	Female gender is associated with a higher prevalence of dysplastic anatomic features such as increased Q angle, femoral anteversion, and patella alta, and thus may result in a higher incidence of patellofemoral conditions, including recurrent patellar dislocation
19.	Rixe et al [19]	2013	Review article	-	Taping and isometric trengthening may be an effective treatment in patients with PFPS, notably in patients with lower body mass index, more severe baseline pain, a larger Q angle, and smaller mean lateral patellofemoral angle
20.	Powers et al. [20]	2002	Prospective study	42	larger Q angle would increase the lateral force vector acting on the patella and may contribute to lateralization of the patella.
21.	Earl et al [21]	2011	Case series	19	exercise-only rehabilitation program focusing on strength training and improving neuromuscular control of the hip and core musculature produces positive patient outcomes, improves the hip and core muscle strength, and reduces the knee abduction moment, which are all related in developing PFPS
22.	Habusta et al. [22]	2022	Statpearls	-	There will be increase in Q angle related to chondromalacia patient
23.	Savelsbergh et al. [23]	1999	Statpearls	-	Coordination of movement is the process of mastering redundant degrees of freedom of the moving organ, in order words, its conversion to a controllable system
24.	Smith-Oricchio et al. [24]	1990	Descriptive observational study	20	Interrater reliability of weight bearing measurements is superior to those of non-weight bearing.
25.	Nara et al. [25]	2022	Control laboratory research	14	Isometric peak torque at 30˚ of knee flexion was lower in the injured limb than in the uninjured limb, but not at 60 ˚and 90˚
26.	Padasala et al [26]	2019	Case control study	100	Increase in Q angle is related to anterior knee pain and it is also found that Long distance runners with large or asymmetric Q-angles may be at greater risk for running injury.
27.	Kaufman et al. [27]	2000	Review article	-	Some of the biomechanical factor such as genu valgum , bone geometry and hamstring flexibility can be modulated by training , equipment or footwear changes.
28.	Jaiyesimi and Jegede [28]	2009	Prospective study	400	Right and left Q angle are not equal in same individual and are higher in women. it is necessary to measured both limb for good result
29.	Rihn et al. [29]	2004	Review article	-	Irrespective of the angle selected, to maintained tibiofemoral joint is essential. Nonsurgical management demands frequent radiographs, especially in the first few weeks, to ensure that the joint remains in the reduced position.
30.	Nguyen et al. [30]	2009	Descriptive cohort study design	218	Greater femoral anteversion and tibiofemoral angle result in greater Q angle, with changes in tibiofemoral angle having a substantially greater impact on the magnitude of the Q angle compared with femoral anteversion
31.	Denızoglu et al. [31]	2019	Descriptive study	90	Q angle is a frontal plane angle where the mediolateral move ments occur and it is also related to the tibifemoral angle, so that greater tibiofemoral angle result in higher Q angle.
32.	Chaudhary et al. [32]	2022	Descriptive observational study	130	Q angle is a better indicator for anterior knee pain than condylar distance. Females in either category; sedentary and sports person, had higher Q angle in comparison to males making them more susceptible to disorders of the patellofemoral joint
33.	Beasley and Vidal [33]	2004	Review	-	Increased Q angles have been considered a risk factor for patellar instability and larger Q angles subject the patella to a larger overall lateral force vector.
34.	Pincivero et al. [34]	2004	Descriptive observational Study	29	QF muscle are at their shortest length when the knee is fully extended and QF muscle-induced anterior tibiofemoral shear force is greatest at more extended knee angles in the open kinetic chain position
35.	Wearing et al. [35]	2006	Review	-	Multifactorial nature of musculoskeletal disorders, it is likely that overweight and obesity may act as a permissive factor in musculoskeletal disease by interacting and potentiating the effects of other risk factors, such as skeletal alignment and muscular deconditioning
36.	Lankhorst et al. [36]	2013	Systematic review	-	Knee hyperextension angle was significantly greater in PFPS patients compared to controls
37.	Van der Worp et al. [37]	2015	Systematic review	-	Analysis of the sex ratios showed that women are at lower risk of running injuries than men. Factors that increased the risk of running-related injuries in women were older age, previous participation in non-axial sports
38.	Sanchez et al. [38]	2014	Descriptive cross- sectional study	62	there are significant differences in the standing position with abducted feet and parallel to the left leg, and symmetry between the lower limbs independent of rotation of limbs in the supine posture and in the supine position there is no asymmetry of the Q angle
39.	Roush et al. [39]	2008	Retrospective study	30	Measurement of Q angle obtained with the goniometer compared with IMAEJ program which is easy to obtain , easy to use, and allows for more permanent digital documentation of the results of Q angle measurement by the clinician.
40.	Power et al. [40]	2000	Observational study	23	The VMO activity could not be shown to be predictive of patellar kinematics illustrates the limitation associated with the use of EMG ratios as indication of patellofemoral joint pathomechanics
41.	Katchburian et al. [41]	2003	Review article	-	Normal patellar tracking remains an elusive goal and it is critical to understanding patellofemoral disorders and indicating treatment appropriately
42.	Delgado-Martinez et al. [42]	2000	Prospective	18	accuracy and reproducibility of imaging methods in the assessment of the patellofemoral joints are essential when planning treatment
43.	CONTARLI and ÖZMEN [43]	2021	Prospective	24	there was no significant relationship between the Q angle and vertical jump height in gymnasts and plays an important role in lower extremity biomechanics, is a widely researched in both athletes and individuals with patellofemoral dysfunction
44.	Hvid and Andersen [44]	1982	Prospective study	29	high Q-angle induces compressive and tensile stress to a point where pathological cartilage change is likely to occur
45.	Olerud and Berg [45]	1984	Prospective study	34	Q angle increases with the shift of foot outward to inward rotation and decreases as the foot shifts from pronation to supination.
46.	Caylor et al. [46]	1993	Descriptive study	20	No significant difference found between the symptomatic and asymptomatic groups as Q angle is not significantly change with 24.3˚ of knee flexion
47.	Tuna et al. [47]	2014	Retrospective study	301	the lateral patellar tilt angle is decreased—therefore, patellar tilt is increased—in patients with chondromalacia patellae
48.	Farrokhi et al. [48]	2011	Controlled laboratory study	10	Baseline reduction in patellar cartilage thickness and patellar cartilage are associated with presence of PFP symptoms.
49.	Magnussen et al. [49]	2014	Systematic review		Risk of recurrent patellar dislocation are low and relatively high risk of persistent feelings of instability may be influenced by the choice of proximal soft tissue procedure at the time of surgery
50.	Caspari [50]	2003	Review article	-	the more widely spaced hips in women explain the finding of higher Q-angles
51.	Saper and Shneider [51]	2014	Review article	-	with increasing knee flexion angles, there would continue to be increased load sharing of the retinaculum and By repairing the lateral release with an iliotibial band rotation flap, the load sharing function of the lateral retinaculum is restored and patellofemoral contact pressures are normalized.
52.	Tecklenburg et al. [52]	2006	Review article	-	Patellofemoral joint has to withstand compression and tension and the patella also serves as a biological lever arm in transmitting the force of the quadriceps muscle
53.	Ariumi et al. [53]	2010	Prospective observational study	45	Both of the extension– Flexion angles were significantly lower genu recurvatum in women than in men; in contrast, no difference was found for adduction–abduction or rotational angles with regard to sex
54.	Nicola and Jewison [54]	2012	Review article	-	Closed kinetic chain through the lower extremities, control of the lumbopelvic mechanism, and overall symmetry of movement has been described well enough that deviations from normal movement can now be associated with specific overuse injuries experienced by runners
55.	Noonan et al. [55]	2022	Review article	-	Rotational deformity is a less common cause of patellar instability than trochlear dysplasia and patella alta, but is an important risk factor for any PFP
56.	Bouffard [56]	1993	Review article	-	Research in adapted physical activity is plagued with a number of particular problems. Among them,frequently noticed ones include the small sample size available for research and the heterogeneity of subjects
57.	Shambaugh et al. [57]	1991	Descriptive study	45	Bilateral weight difference and Q angle is most important measurement within the player to predict injury status in basketball player
58.	McConnell [58]	1986	Review article		An abnormally high Q angle indicates lateral pull of the patella in the trochlear groove of the femur and a mechanism of articular cartilage wear and tear.
59.	Espandar et al. [59]	2009	Review article		Angular deformities of the lower limbs are common during childhood and usually make serious concern for the parents and these deformities represent normal variations of the growth and development of the child and needs no treatment except for observation and reassurance of the parents
60.	Chantraine A [60]	1985	Prospective observational study	81	Stress and strain imposed on a joint during growth and adolescence through intensive practice of sport may contribute to such deformity.
61.	Brooks and Gross [61]	1995	Review article	-	Genu varum is a relatively common finding in children and it also accompany systemic conditions, such as achondroplasia, vitamin D–resistant rickets, renal osteodystrophy, and osteogenesis mperfect—all of which can result in short stature
62.	Arazi et al. [62]	2001	Normative study	590	the intraexaminer variability was found within the average range of other studies and angle interpreted photographically closely approximates true TF angle.
63.	Lun et al. [63]	2004	observational	87	static biomechanical alignment measurements of the lower limbs are not related to lower limb injury except patellofemoral pain syndrome
64.	Messier et al. [64]	1991	case control study	36	An increased in Q angle may be caused by lateral displacement of tibial tuberosity, increase femoral anteversion, genu valgum or external tibial torsion.
65.	Taunton et al. [65]	2002	A retrospective case control analysis		The knee was the most common injury location and five most common injuries were PFPS, ITBFS, plantar fasciitis, meniscal injuries, and patellar tendinopathy. In addition, certain injuries occurred with a statistically significant higher frequency in one sex than the other.
66.	Van Mechelen [66]	1992	Review article		Causes of running injuries is limited to musculoskeletal injuries, the most common running injuries. With regard to early recognition of overuse injuries a runner should be taught to listen and to respect ‘the language of his body’ and reduce or temporarily stop, rather than to continue or increase, running when suffering from pain or stiffness of joints and tendons as a result of running.
67.	Wen et al. [67]	1998	A prospective cohort study	355	Runners are often advised to alternate more than one pair of shoes and to change new shoes frequently to prevent knee injuries
68.	Bhalara et al. [68]	2013	Observational study	100	With increase in the age there was significant increase in values of Q angle in children of both the sexes and there was no significant difference between the boys and girls in Q angle in all ages.
69.	Çankaya et al. [69]	2020	Observational study	599	Positional changes and weight bearing on limbs did not cause any change in knee position in healthy children and the decrease in quadriceps angle in this age group is due to growth rate asymmetry between the femur shaft and pelvic diameter.

## Conclusions

The Q angle's enigmatic nature is caused by several things, including the acceptance of what appears to be a widely held but incorrect assumption and the lack of methodological clarity. Currently, much literature is found which helps us conclude that excessive increase and decrease in Q angle affects knee extensor mechanism and causes various pathologies with the increase in age. While women show a higher mean Q angle value than men, the underlying causes of the difference are not immediately apparent. The widely held belief that women have wider hips than men and that Q angles are bilaterally symmetrical is not corroborated by empirical data. A new strategy for studying the Q angle must take the place of these antiquated presumptions. It is essential to use analytical methods that consider the similarities and differences between group and individual bilateral Q angle measurements and a consistent approach that accounts for all elements of the measurement procedure.

## References

[1] Loudon JK (2016). Biomechanics and pathomechanics of the patellofemoral joint. Int J Sports Phys Ther.

[2] Boling MC, Padua DA, Marshall SW, Guskiewicz K, Pyne S, Beutler A (2009). A prospective investigation of biomechanical risk factors for patellofemoral pain syndrome: the Joint Undertaking to Monitor and Prevent ACL Injury (JUMP-ACL) cohort. Am J Sports Med.

[3] Mizuno Y, Kumagai M, Mattessich SM, Elias JJ, Ramrattan N, Cosgarea AJ (2001). Q-angle influences tibiofemoral arid patellofemoral kinematics. J Orthop Res.

[4] Huston LJ, Greenfield ML, Wojtys EM (2000). Anterior cruciate ligament injuries in the female athlete: potential risk factors. Clin Orthop Relat Res.

[5] Frank JB, Jarit GJ, Bravman JT, Rosen JE (2007). Lower extremity injuries in the skeletally immature athlete. J Am Acad Orthop Surg.

[6] Kim HK, Parikh S (2022). Patellofemoral instability in children: imaging findings and therapeutic approaches. Korean J Radiol.

[7] Covassin T, Crutcher B, Bleecker A, Heiden EO, Dailey A, Yang J (2014). Postinjury anxiety and social support among collegiate athletes: a comparison between orthopaedic injuries and concussions. J Athl Train.

[8] Hilibrand MJ, Hammoud S, Bishop M, Woods D, Fredrick RW, Dodson CC (2015). Common injuries and ailments of the female athlete; pathophysiology, treatment and prevention. Phys Sportsmed.

[9] Dixit S, Difiori JP, Burton M, Mines B (2007). Management of patellofemoral pain syndrome. Am Fam Physician.

[10] Grelsamer RP (2000). Patellar malalignment. J Bone Joint Surg.

[11] Percy EC, Strother RT (1985). Patellalgia. Phys Sportsmed.

[12] van Gent RN, Siem D, van Middelkoop M, van Os AG, Bierma-Zeinstra SM, Koes BW (2007). Incidence and determinants of lower extremity running injuries in long distance runners: a systematic review. Br J Sports Med.

[13] Murray TF, Dupont JY, Fulkerson JP (1999). Axial and lateral radiographs in evaluating patellofemoral malalignment. Am J Sports Med.

[14] Esculier JF, Maggs K, Maggs E, Dubois B (2020). A contemporary approach to patellofemoral pain in runners. J Athl Train.

[15] Patel DR, Yamasaki A, Brown K (2017). Epidemiology of sports-related musculoskeletal injuries in young athletes in United States. Transl Pediatr.

[16] Fulkerson JP (2002). Diagnosis and treatment of patients with patellofemoral pain. Am J Sports Med.

[17] Steinberg N, Tenenbaum S, Hershkovitz I, Zeev A, Siev-Ner I (2017). Lower extremity and spine characteristics in young dancers with and without patellofemoral pain. Res Sports Med.

[18] Waterman BR, Belmont PJ Jr, Owens BD (2012). Patellar dislocation in the United States: role of sex, age, race, and athletic participation. J Knee Surg.

[19] Rixe JA, Glick JE, Brady J, Olympia RP (2013). A review of the management of patellofemoral pain syndrome. Phys Sportsmed.

[20] Powers CM, Chen PY, Reischl SF, Perry J (2002). Comparison of foot pronation and lower extremity rotation in persons with and without patellofemoral pain. Foot Ankle Int.

[21] Earl JE, Hoch AZ (2011). A proximal strengthening program improves pain, function, and biomechanics in women with patellofemoral pain syndrome. Am J Sports Med.

[22] Habusta SF, Coffey R, Ponnarasu S, Mabrouk A, Griffin EE (2021). Chondromalacia Patella. Habusta, S.F. and Coffey, R. and Ponnarasu, S. and patella, Griffin E.E.Chondromalacia.

[23] Savelsbergh G, Wimmers R, Kamp JV, Davids K (1999). The development of movement control and coordination. Current Issues in Developmental Psychology.

[24] Smith-Oricchio K, Harris BA (1990). Interrater reliability of subtalar neutral, calcaneal inversion and eversion. J Orthop Sports Phys Ther.

[25] Nara G, Samukawa M, Oba K, Koshino Y, Ishida T, Kasahara S, Tohyama H (2022). The deficits of isometric knee flexor strength in lengthened hamstring position after hamstring strain injury. Phys Ther Sport.

[26] Padasala M (2019). Relationship between bilateral quadriceps angle and anterior knee pain and its association with knee injury in long distance runners. J Sports Med.

[27] Kaufman KR, Brodine S, Shaffer R (2000). Military training-related injuries: surveillance, research, and prevention. Am J Prev Med.

[28] Jaiyesimi AO, Jegede OO (2009). Influence of gender and leg dominance on q-angle among young adult Nigerians. S Afr J Physiother.

[29] Rihn JA, Groff YJ, Harner CD, Cha PS (2004). The acutely dislocated knee: evaluation and management. J Am Acad Orthop Surg.

[30] Nguyen AD, Boling MC, Levine B, Shultz SJ (2009). Relationships between lower extremity alignment and the quadriceps angle. Clin J Sport Med.

[31] Denızoglu Kulli H, Yeldan I, Yildirim NU (2019). Influence of quadriceps angle on static and dynamic balance in young adults. J Back Musculoskelet Rehabil.

[32] Chaudhary S, Jain SK, Sharma N, Bhatnagar S (2022). Analysis of predictors affecting biomechanical function of the knee joint and its relation to anterior knee pain. Cureus.

[33] Beasley LS, Vidal AF (2004). Traumatic patellar dislocation in children and adolescents: treatment update and literature review. Curr Opin Pediatr.

[34] Pincivero DM, Salfetnikov Y, Campy RM, Coelho AJ (2004). Angle- and gender-specific quadriceps femoris muscle recruitment and knee extensor torque. J Biomech.

[35] Wearing SC, Hennig EM, Byrne NM, Steele JR, Hills AP (2006). Musculoskeletal disorders associated with obesity: a biomechanical perspective. Obes Rev.

[36] Lankhorst NE, Bierma-Zeinstra SM, van Middelkoop M (2013). Factors associated with patellofemoral pain syndrome: a systematic review. Br J Sports Med.

[37] van der Worp MP, ten Haaf DS, van Cingel R, de Wijer A, Nijhuis-van der Sanden MW, Staal JB (2015). Injuries in runners; a systematic review on risk factors and sex differences. PLoS One.

[38] Sanchez HM, Sanchez EG, Baraúna MA, Canto RS (2014). Evaluation of Q angle in differents static postures. Acta Ortop Bras.

[39] Roush JR, Bustillo K, Low E (2008). Measurement error between a goniometer and the NIH ImageJ program for measuring quadriceps angle. Internet J Allied Health Sci Pract.

[40] Powers CM (2000). Patellar kinematics, part I: the influence of vastus muscle activity in subjects with and without patellofemoral pain. Phys Ther.

[41] Katchburian MV, Bull AM, Shih YF, Heatley FW, Amis AA (2003). Measurement of patellar tracking: assessment and analysis of the literature. Clin Orthop Relat Res.

[42] Delgado-Martínez AD, Rodríguez-Merchán EC, Ballesteros R, Luna JD (2000). Reproducibility of patellofemoral CT scan measurements. Int Orthop.

[43] Contarli N, Özmen T (2021). Relationship between Q angle, dynamic balance and vertical jump height in gymnasts. Çanakkale Onsekiz Mart Üniversitesi Spor Bilimleri Dergisi.

[44] Hvid I, Andersen LI (1982). The quadriceps angle and its relation to femoral torsion. Acta Orthop Scand.

[45] Olerud C, Berg P (1984). The variation of the Q angle with different positions of the foot. Clin Orthop Relat Res.

[46] Caylor D, Fites R, Worrell TW (1993). The relationship between quadriceps angle and anterior knee pain syndrome. J Orthop Sports Phys Ther.

[47] Tuna BK, Semiz-Oysu A, Pekar B, Bukte Y, Hayirlioglu A (2014). The association of patellofemoral joint morphology with chondromalacia patella: a quantitative MRI analysis. Clin Imaging.

[48] Farrokhi S, Colletti PM, Powers CM (2011). Differences in patellar cartilage thickness, transverse relaxation time, and deformational behavior: a comparison of young women with and without patellofemoral pain. Am J Sports Med.

[49] Magnussen RA, De Simone V, Lustig S, Neyret P, Flanigan DC (2014). Treatment of patella alta in patients with episodic patellar dislocation: a systematic review. Knee Surg Sports Traumatol Arthrosc.

[50] Caspari R (2003). From types to populations: a century of race, physical anthropology, and the American Anthropological Association. Am Anthropol.

[51] Saper MG, Shneider DA (2014). Diagnosis and treatment of lateral patellar compression syndrome. Arthrosc Tech.

[52] Tecklenburg K, Dejour D, Hoser C, Fink C (2006). Bony and cartilaginous anatomy of the patellofemoral joint. Knee Surg Sports Traumatol Arthrosc.

[53] Ariumi A, Sato T, Kobayashi K, Koga Y, Omori G, Minato I, Endo N (2010). Three-dimensional lower extremity alignment in the weight-bearing standing position in healthy elderly subjects. J Orthop Sci.

[54] Nicola TL, Jewison DJ (2012). The anatomy and biomechanics of running. Clin Sports Med.

[55] Noonan B, Cooper T, Chau M, Albersheim M, Arendt EA, Tompkins M (2022). Rotational deformity—when and how to address femoral anteversion and tibial torsion. Clin Sports Med.

[56] Bouffard M (1993). The perils of averaging data in adapted physical activity research. Adapt Phys Activ Q.

[57] Shambaugh JP, Klein A, Herbert JH (1991). Structural measures as predictors of injury basketball players. Med Sci Sports Exerc.

[58] McConnell J (1986). The management of chondromalacia patellae: a long term solution. Aust J Physiother.

[59] Espandar R, Mortazavi SM, Baghdadi T (2010). Angular deformities of the lower limb in children. Asian J Sports Med.

[60] Chantraine A (1985). Knee joint in soccer players: osteoarthritis and axis deviation. Med Sci Sports Exerc.

[61] Brooks WC, Gross RH (1995). Genu varum in children: diagnosis and treatment. J Am Acad Orthop Surg.

[62] Arazi M, Oğün TC, Memik R (2001). Normal development of the tibiofemoral angle in children: a clinical study of 590 normal subjects from 3 to 17 years of age. J Pediatr Orthop.

[63] Lun V, Meeuwisse WH, Stergiou P, Stefanyshyn D (2004). Relation between running injury and static lower limb alignment in recreational runners. Br J Sports Med.

[64] Messier SP, Davis SE, Curl WW, Lowery RB, Pack RJ (1991). Etiologic factors associated with patelofemoral pain in runners. Med Sci Sports Exerc.

[65] Taunton JE, Ryan MB, Clement DB, McKenzie DC, Lloyd-Smith DR, Zumbo BD (2002). A retrospective case-control analysis of 2002 running injuries. Br J Sports Med.

[66] van Mechelen W (1992). Running injuries: a review of the epidemiological literature. Sports Med.

[67] Wen DY, Puffer JC, Schmalzried TP (1998). Injuries in runners: a prospective study of alignment. Clin J Sport Med.

[68] Bhalara A, Talsaniya D (2013). Q angle in children population aged between 7 to 12 years. Int J Health Sci Res.

[69] Çankaya T, Dursun Ö, Davazlı B, Toprak H, Çankaya H, Alkan B (2020). Assessment of quadriceps angle in children aged between 2 and 8 years. Turk Pediatri Ars.

